# Multi loop snare technique for difficult inferior vena cava filter retrievals

**DOI:** 10.1186/s42155-018-0042-0

**Published:** 2018-12-20

**Authors:** Arash Najafi, Katerina Koulia, Philippe Aubert, Christoph A. Binkert

**Affiliations:** Institute of Radiology and Nuclear Medicine, Kantonsspital Winterthur, Brauerstrasse 15, 8405 Winterthur, Switzerland

**Keywords:** Pulmonary embolism, IVC filter, Venous intervention, Loop snare technique

## Abstract

**Introduction:**

Use of optional vena cava filters has steadily increased. In the majority of cases removal is successful using standard techniques. In cases of tilting and migration of the filter however, more advanced techniques are necessary. The “loop-snare” technique has been described for such cases. Difficulties arise when the loop starts to slip around the legs and arms of the filter.

**New technique:**

We present an improved loop-snare technique which allows to retrieve IVC filters when the simple loop-snare technique fails. We used additional loops, in one case one additional loop in another case two additional loops around the filter tip which allowed successful retrieval. The additional loops were created with a reversed shaped catheter. All guidewires were then engaged with a snare and pulled into a large sheath. The additional loops stabilize the tip and the filter can be pulled into the sheath.

**Conclusion:**

The “multiple-loop-snare” technique is a refinement of the previously described “single loop-snare” technique and can be used when one loop fails.

## Introduction

Optional inferior vena cava (IVC) filters are used for patients with thromboembolic disease in whom anticoagulation is temporarily contraindicated. Additionally, they can be placed prophylactically in patients with high risk of thromboembolic disease, e.g. before bariatric or spine surgery or after polytrauma. With the possibility to retrieve IVC filters the use of cava filters has increased in the past years (Stein et al., 2011).

Due to potential complications of indwelling IVC filters, their removal is recommended when filtration is no longer needed. Although optional filters can be left in place as permanent filters, retrieval is necessary in certain instances like severe tilt or penetration outside the IVC. Standard retrieval techniques have high technical success rates (75–100%) (Doody et al., 2009; Rubenstein et al., 2007; Iliescu & Haskal, 2011) and few complications. In case of migration or tilt of the filter however, removal can become challenging. The main reasons for unsuccessful retrievals are filter tilt with potential endothelialization and trapped emboli within the filter (Doody et al., 2009; Rubenstein et al., 2007; Iliescu & Haskal, 2011; Kuo et al., 2011; Kassavin & Constantinopoulos, 2011; Kuo & Cupp, 2010; Binkert et al., 2005). In such cases advanced retrieval techniques have been described. Examples include the use of the Recovery Cone (Miller et al., 2006), cone over the wire technique (Kassavin & Constantinopoulos, 2011), use of a tip-deflecting wire (Hagspiel et al., 2004), the use of a curved sheath (Yamagami 2005) balloon displacement technique (Lynch, 2009), double wire restraining or “sandwich” technique (Owens et al., 2011), and dissection techniques with forceps or laser (Kuo & Cupp, 2010; Stavropoulos et al., 2006; Burke et al., 2007; McBride et al., 2010). However, not all techniques are equally suited for the same situation, depending on the position and tilt of the filter and dwelling time.

The “loop-snare” technique, which was previously described by Rubenstein et al. (Rubenstein et al., 2007) for filters that are tilted against the IVC wall, enables the operator to remove the tip from the caval wall and consequently retrieve the IVC filter. The concept is as followed: a reverse curve catheter is positioned below the filter tip and used to direct a guidewire backwards in a “U-turn” fashion. The wire is then snared above the filter thereby creating a loop around the filter tip. The sheath is then advanced towards the tip of the filter and traction is applied to both ends of the wire to pull the filter tip away from the vessel wall into the sheath.

We describe a multi-loop-snare technique which was applied twice successfully after a failed attempt with one loop. Instead of one loop, two respectively three loops were created around the filter tip for successful removal. Both cases were performed under local anesthesia.

## Cases

### Case 1

A 31-year-old female patient with symptomatic postpartum deep venous thrombosis of the right leg up to the distal part of the inferior vena cava prophylactically received a suprarenal IVC filter (Recovery G2; Bard Peripheral Vascular) before surgical thrombectomy. Suprarenal position had to be chosen due to short distance between thrombus in the distal IVC and inflow of renal veins. Surgical access was established via venotomy of the common femoral vein and thrombectomy was performed using an occlusion balloon. In a follow-up CT it was noted that the filter had dislodged and tilted towards the right IVC wall with its legs piercing the contralateral wall and extending into the left renal vein, likely during surgical thrombectomy. Because of re-thrombosis despite continued oral anticoagulation with Warfarin, catheter-directed thrombolysis including stenting of the common iliac veins and the IVC was performed. No filter retrieval was attempted because of large amount of clot within the filter.

After 5 weeks of Warfarin therapy the patient was scheduled for retrieval which was considered mandatory due to the displaced and tilted filter with penetrating legs. After introduction of a 14-F sheath (Cook Medical, Bloomington, Indiana, USA), a SOS catheter (Omni 2, 5F, 80 cm, Angiodynamics, New York, USA) together with a Bentson wire (260 cm, Cook Medical, Bloomington, Indiana, USA) and EN Snare (7F, 18–30 mm, Merit Medical, Utah, USA) were used to create a loop. The single loop-snare technique was attempted several times without success because the loop kept slipping around the filter legs. In order to stabilize the loop a second loop using the same technique was formed around the filter tip. Withtwo loops around the filter tip, the filter was successfully pulled away from the wall and into the 14F sheath.

A follow-up venography was unremarkable. Follow-up ultrasound after 6 months showed patent IVC with regular flow. Genetic tests revealed a heterozygous Factor V Leiden mutation and Warfarin therapy was continued for at least 2 years, after which the patient was lost to follow-up.

### Case 2

**Fig. 1:**
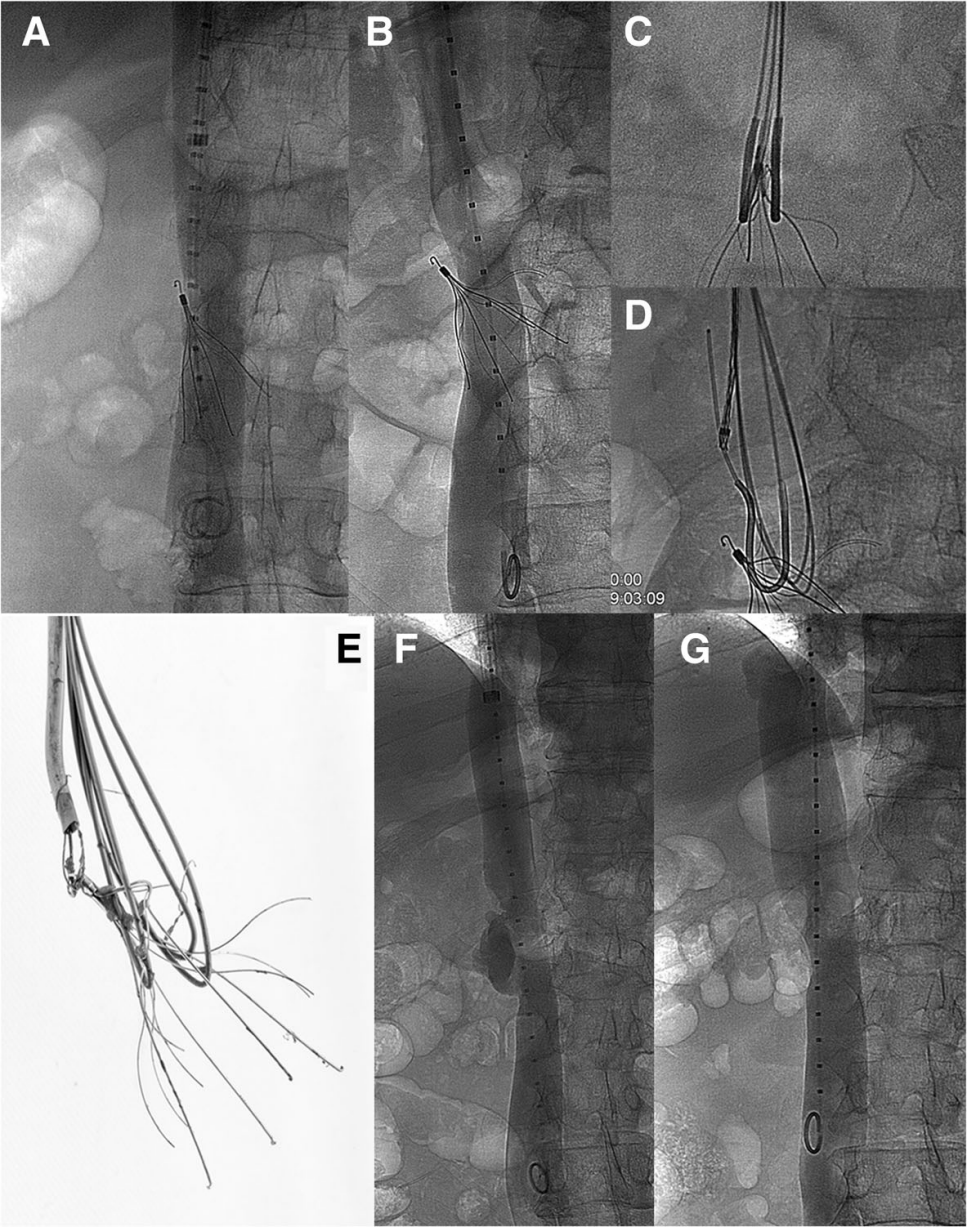
**a** Venography of IVC before first removal attempt shows a tilted Celect filter with one leg protruding outside the IVC. **b** A repeat venography 8 years later shows a more tilted filter with a deeply embedded filter tip and two legs outside the IVC. **c** A lateral view confirms one loop on each side of the filter tip. **d** A third loop was created using a reversed shape catheter an exchange length guidewire and a snare. **e** Photography after explantation illustrates the three loops around different legs and arms of the filter. **f** Venography immediately after filter removal shows a contrast pocket at the location of the former filter tip location. **g** A control venography 6 weeks later reveals a smooth IVC with only a mild stenosis at the level of previous filter location

A 50 year old male received an infrarenal IVC filter (Celect, Cook Medical, Bloomington, Indiana, USA) before orthopedic surgery of the lower extremities due to prior history of Factor V Leiden mutation with several previous episodes of deep vein thromboses. After successful surgery and resumption of Warfarin a filter retrieval was attempted 74 days later. Venography revealed a tilted filter with the tip towards the right IVC wall. Standard technique didn’t seem feasible, therefore a loop-snare technique was attempted using the same equipment and technique as mentioned in Case 1. Despite a successful loop around the filter tip, filter retrieval was unsuccessful. At that time the filter was left in place because of an only moderate tilt and only one leg protruding outside the IVC (Fig. 1a). Warfarin was continued for at least a year and then stopped due to repeated anorectal bleeding episodes.

During a CT for macrohematuria more than 8 years later a severe tilt of the filter was seen with two legs around the aorta and one leg eroding the bone of a vertebral body. In a multidisciplinary board the decision for another retrieval attempt was made. 3146 days after implantation the patient was scheduled for a second attempt. Expecting a difficult retrieval an 18-F sheath (Cook Medical, Bloomington, Indiana, USA) was inserted into the right jugular vein. Venography confirmed a tilted filter with the tip deeply embedded into the IVC wall and two legs protruding outside the IVC (Fig. 1b). Two loops were formed around the filter tip using the same instruments previously described: two reversed shape SOS-catheters (Omni 2, 5F, 80 cm, Angiodynamics, New York, USA) were placed below the filter and two exchange length Bentson wires (260 cm, Cook Medical, Bloomington, Indiana, USA) were navigated on both sides of the filter tip. Above the tip the wires were snared. A second view confirmed one loop on each side of the filter tip (Fig. 1c). During traction both loops started to slip away from the tip, therefore a third loop was created (Fig. 1d) again using a Bentson wire. With 3 loops around the filter tip, the filter could be removed from the wall and finally pulled into the 18F sheath. Configuration of the 3 loops around different filter struts was documented after retrieval (Fig. 1e). During final traction the patient expressed stinging pain in the back. Post-interventional venography showed a large contrast pocket was visible at the location of the embedded filter tip (Fig. 1f) without a true extravasation. An immediate CT did not show extravasation or retroperitoneal hematoma. Because the patient was hemodynamically stable no further treatment was undertaken.

A venography 6 weeks later showed a regular shape of the IVC with only minimal narrowing (Fig. 1g).

## Conclusion

Retrieval of tilted and/or embedded IVC filters can be very difficult. The goal is to extract the filter with minimal risk for complications (e.g. dissection, rupture, thrombosis, filter breakdown etc.) The simple “loop-snare” technique has been described previously and has a high rate of success. Sometimes however, one loop may slip around the filter tip preventing a retrieval. In such cases a second or third loop can help stabilize the filter enabling the operator to pull the filter away from the wall and into a large sheath. It is important to use different fulcrums to create a more stable situation.

The “multi-loop-snare” technique is an atraumatic filter retrieval technique that does not include sharp instrumentation and/or dissection techniques which could lead to direct injury of the IVC. As shown in case 2, even deeply embedded filters can be retrieved successfully. Irregularities of the IVC can be observed which in our case healed over time without sequelae. Similar “healing” of the IVC has been previously described (Binkert et al., 2007). Additionally, the procedure can be abandoned at any time without risking entanglement of the wires to the filter. Another advantage of this technique is the single access site in comparison to other techniques. We recommend the insertion of a large sheath (18F) in order to accommodate the dual or triple loop approach. A potential downside of the increased traction force using 2 or 3 instead of a single loop might be a higher risk of filter fracture due to greater localized torque at the fulcrums. This however has not been described in the literature with this technique.

In conclusion, the multi-loop-snare technique is a refined version of the single loop-snare technique for difficult IVC filter retrievals. The multiple-loop-snare technique allows a better alignment of the filter tip leading to a better control and traction during retrieval. It seems that adding loops can further increase the success rate of retrievals.

## Abbreviation

IVC: Inferior vena cava
